# Comparison of enzymatic reactivity of corn stover solids prepared by dilute acid, AFEX™, and ionic liquid pretreatments

**DOI:** 10.1186/1754-6834-7-71

**Published:** 2014-05-13

**Authors:** Xiadi Gao, Rajeev Kumar, Seema Singh, Blake A Simmons, Venkatesh Balan, Bruce E Dale, Charles E Wyman

**Affiliations:** 1 BioEnergy Science Center (BESC), Oak Ridge National Laboratory, Oak Ridge, TN 37831, USA; 2 Department of Chemical and Environmental Engineering, Bourns College of Engineering, University of California (UCR), Riverside, CA 92521, USA; 3 Center for Environmental Research and Technology (CE-CERT), Bourns College of Engineering, University of California, Riverside, CA 92507, USA; 4 Deconstruction Division, Joint BioEnergy Institute (JBEI), Emeryville, CA 94608, USA; 5 Sandia National Laboratories, Livermore, CA 94551, USA; 6 DOE Great Lakes Bioenergy Research Center (GLBRC), Michigan State University, East Lansing, MI 48824, USA; 7 Biomass Conversion Research Laboratory, Department of Chemical Engineering and Materials Science, Michigan State University, 3815 Technology Boulevard, MBI Building, Lansing, MI 48910, USA

**Keywords:** Corn stover, Enzyme adsorption, Cellulase, Oligomers, Pretreatment, Hydrolysis

## Abstract

**Background:**

Pretreatment is essential to realize high product yields from biological conversion of naturally recalcitrant cellulosic biomass, with thermochemical pretreatments often favored for cost and performance. In this study, enzymatic digestion of solids from dilute sulfuric acid (DA), ammonia fiber expansion (AFEX™), and ionic liquid (IL) thermochemical pretreatments of corn stover were followed over time for the same range of total enzyme protein loadings to provide comparative data on glucose and xylose yields of monomers and oligomers from the pretreated solids. The composition of pretreated solids and enzyme adsorption on each substrate were also measured to determine. The extent glucose release could be related to these features.

**Results:**

Corn stover solids from pretreatment by DA, AFEX, and IL were enzymatically digested over a range of low to moderate loadings of commercial cellulase, xylanase, and pectinase enzyme mixtures, the proportions of which had been previously optimized for each pretreatment. Avicel® cellulose, regenerated amorphous cellulose (RAC), and beechwood xylan were also subjected to enzymatic hydrolysis as controls. Yields of glucose and xylose and their oligomers were followed for times up to 120 hours, and enzyme adsorption was measured. IL pretreated corn stover displayed the highest initial glucose yields at all enzyme loadings and the highest final yield for a low enzyme loading of 3 mg protein/g glucan in the raw material. However, increasing the enzyme loading to 12 mg/g glucan or more resulted in DA pretreated corn stover attaining the highest longer-term glucose yields. Hydrolyzate from AFEX pretreated corn stover had the highest proportion of xylooligomers, while IL produced the most glucooligomers. However, the amounts of both oligomers dropped with increasing enzyme loadings and hydrolysis times. IL pretreated corn stover had the highest enzyme adsorption capacity.

**Conclusions:**

Initial hydrolysis yields were highest for substrates with greater lignin removal, a greater degree of change in cellulose crystallinity, and high enzyme accessibility. Final glucose yields could not be clearly related to concentrations of xylooligomers released from xylan during hydrolysis. Overall, none of these factors could completely account for differences in enzymatic digestion performance of solids produced by AFEX, DA, and IL pretreatments.

## Background

Lignocellulosic biomass, including agricultural and forestry residues and herbaceous and woody crops [1], provides the only sustainable resource with potential for large-scale and low-cost production of liquid fuels and organic chemicals that are currently produced from dwindling and nonrenewable fossil resources that are major contributors to greenhouse gas emissions [1,2]. Enzymatic hydrolysis is a key step in the biological conversion of lignocellulosic biomass into fuels and chemicals, with the high product yields important to commercial success [1-5]. Endoglucanases, exoglucanases, and β-glucosidase as well as supplementary enzymes such as xylanases and β-xylosidase are generally required to complete enzymatic hydrolysis effectively and efficiently [6-10]. However, to realize the high yields vital to commercial success of enzymatic conversion [11], most cellulosic biomass must be pretreated prior to enzymatic hydrolysis, and the choice of pretreatment not only affects enzymatic digestion performance but impacts upstream and downstream processing as well [1,12]. To overcome the natural recalcitrance of cellulosic biomass, several biological, chemical, thermochemical, and physical pretreatment methods have been applied, but thermochemical pretreatments are often preferred due to a more favorable combination of capital costs, operating costs, and performance [12].

Among thermochemical pretreatments, hemicellulose or lignin removal and/or alternation by dilute acids, with just hot water, or base promise reasonable costs [11,13,14]. In particular, dilute sulfuric acid (DA) and ammonia fiber expansion (AFEX™) pretreatments are currently among the most promising from a combined cost and performance perspective [1]. DA and hydrothermal pretreatments effectively remove and recover as sugars a large portion of hemicellulose as well as disrupting and dislocating lignin, while increasing cellulose digestibility [15-17]. The AFEX process pretreats biomass with anhydrous liquid ammonia at high pressure and moderate to high temperatures. Following pretreatment for a given time, the pressure is rapidly released resulting in biomass structure disruption and partial cellulose decrystallization that presumably enhance cellulose digestibility [18-20]. Lately, certain ionic liquids (ILs) such as the IL 1-ethyl-3-methylimidazolium acetate have been employed for pretreatment followed by addition of an anti-solvent to precipitate biomass [21]. Such ILs remove most of the lignin from biomass and disrupt the native cellulose crystalline structure and hydrogen networks to form cellulose II, thus reducing biomass recalcitrance [22-24].

Various biomass physicochemical changes resulting from the action of different leading pretreatments have been reported to enhance cellulose enzymatic digestibility, such as surface area, pore volume, hemicellulose removal, lignin removal and/or dislocation, crystallinity reduction, and reduced cellulose degree of polymerization (DP) [25-31]. Of these many possible factors, hemicellulose removal and lignin dislocation are mainly credited with enhancing digestion by DA pretreatment [16,17]. Creation of pore structures in biomass and disruption of lignin-carbohydrate complex (LCC) linkages enhance digestion for AFEX pretreatment [32,33]. Increased surface roughness, reduced cellulose crystallinity, and expansion or even a transformation of the cellulose lattice have been reported to account for the beneficial effects of IL pretreatments on biomass [22,34].

With several pretreatments being effective in achieving high sugar yields, it is vital to develop comparative data on sugar release from enzymatic hydrolysis of solids produced by each to facilitate identification of promising technologies. It is also important to measure key features influenced by each pretreatment to determine if enzymatic digestion can be related to any common characteristics, especially at the low enzyme loadings needed to be commercially viable [35]. In line with these objectives, researchers from the University of California, Riverside (UCR; Riverside, CA, USA), supported by the BioEnergy Science Center (BESC; Oak Ridge, TN, USA), Michigan State University (East Lansing, MI, USA) supported by the Great Lakes Bioenergy Research Center (GLBRC; Michigan State University), and the Joint BioEnergy Institute (JBEI; Emeryville, CA, USA) collaborated to better understand how biomass pretreatments by AFEX, DA, and IL with much different deconstruction patterns impact glucose and xylose release from enzymatic digestion over a wide range of enzyme loadings. To provide comparative information, a single source of corn stover was used as the feedstock for all three pretreatments, and one source of commercial cellulases and accessory enzymes was applied to the pretreated solids. In addition to sugar release data, changes in composition of solids and enzyme adsorption capacity of pretreated biomass solids were also measured to determine if they could help explain performance differences. Other papers by this team are in progress to examine changes in other features of pretreated biomass and more fully follow key features that could account for the performance differences reported here.

## Results and discussion

### Compositional analysis of DA, AFEX, and IL pretreated corn stover

Table 1 summarizes the pretreatment conditions applied, compositions of raw and pretreated corn stover solids, and the amount of xylan and lignin removed by each pretreatment. It can be seen that DA removed about 87% of the xylan, resulting in solids with a very low xylan content of 6.5% and increased glucan content of 59.1%. AFEX pretreatment of corn stover, as a dry to dry process, did not solubilize much of the biomass during pretreatment, resulting in a negligible compositional change. However, IL pretreatment removed >90% of the lignin and >20% of the xylan originally in corn stover, resulting in solids with a lignin content of only 2.7% and glucan and xylan contents of 46.9% and 29.8%, respectively. These changes in composition are consistent with results from previously reported studies [31,36,37].

**Table 1:** Pretreatment conditions, corresponding solids compositions, and component removals for pretreatment of corn stover by dilute acid (DA), ammonia fiber expansion (AFEX™), and ionic liquid (IL)

	**Pretreatment**			
	**None**	**DA**	**AFEX**	**IL**
**Pretreatment conditions**				
Chemicals	NA	Dilute sulfuric acid	Anhydrous ammonia	1-ethyl‒3‒methyl‒imidazoliumacetate
Loadings	NA	0.5% wt	1:1 (Biomass: NH_3_)	1:9 (Biomass: IL)
L/S ratio	NA	9:1	1:1	9:1
Temperature (°C)	NA	160	140	140
Time (min)	NA	20	15	180
**Component (%)**				
Glucan	33.4	59.1	33.5	46.9
Xylan	24.9	6.5	24.8	29.8
Arabinan	3.7	3.6	3.3	0.3
Lignin (AIL)	17.2	32.2	12.2	2.7
Component removal (%)*				
Solid		51.0	0	36.0
Xylan	-	87.0	0.4	23.4
Lignin	-	8.2	2.8	89.9

*Solid removal (%) = 100% × (1- Total solid after pretreatment (g)/Total solid before pretreatment (g)).

Xylan removal (%) = 1- (%Xylan in pretreated solids / % Xylan in raw biomass) × Solid yield.

Lignin removal (%) = 1- (%Lignin in pretreated solids / % Lignin in raw biomass) × Solid yield.

L/S - liquid to solid.

### Enzymatic hydrolysis of pretreated corn stover solids and model compounds

#### Glucose yields from enzymatic digestion of pretreated solids

Enzymatic digestion was performed at solids loading of 1% (w/w) glucan to allow focus on how pretreatments impacted substrate digestion and avoid confusion by end-product inhibition of the enzymes; this approach is consistent with the National Renewable Energy Laboratory (NREL) Laboratory Analytical Procedure (LAP) [38]. Enzyme loadings of 3, 6, 12, and 30 mg of total protein/g glucan in the raw biomass were applied at ratios of cellulase (Cellic® CTec2, Novozymes North America, Inc, Franklinton, NC, USA), hemicellulase (Cellic® HTec2, Novozymes North America, Inc), and pectinase (Multifect® Pectinase, DuPont™ Genencor® Science, DuPont Industrial Biosciences, Palo Alto, CA, USA), as shown in Table 2, that GLBRC had previously shown to give the highest sugar release from pretreated solids using their high-throughput microplate-based hydrolysis method [39]. Although different enzyme mixtures were required to achieve the highest sugar yields in enzymatic hydrolysis of solids from each pretreatment, the total mass loadings of the enzyme combinations applied were kept the same. In addition, optimal ratios of enzyme components in the mixtures were not simply related to the composition of the pretreated solids. For example, DA pretreated corn stover had negligible amounts of xylan, but substitution of hemicellulases for some of the cellulase still improved yields, possibly due to synergies or other effects among the enzymes [40].

**Table 2:** Optimized enzyme mixtures on protein mass percents for DA, AFEX, and IL pretreated corn stover as determined by GLBRC with their high-throughput system

**Pretreatment**	**Cellic® CTec2**	**Cellic® HTec2**	**Multifect® Pectinase**
Dilute acid	67%	33%	0
AFEX	67%	16.5%	16.5%
Ionic liquid	39%	33%	28%

All enzymatic hydrolysis reactions were conducted at 50°C for time periods up to 120 hours. Sugar release is reported in terms of the glucose and xylose yield calculations described in the Materials and methods section, in which the amount of either sugar actually released is divided by the maximum amount of the respective sugar that could be released from a particular pretreated solid including the appropriate factors to account for mass changes in hydrolysis. The glucose yield calculation includes monomeric glucose plus cellobiose, while the xylose yield only takes into account the monomeric xylose released.

**Figure 1:**
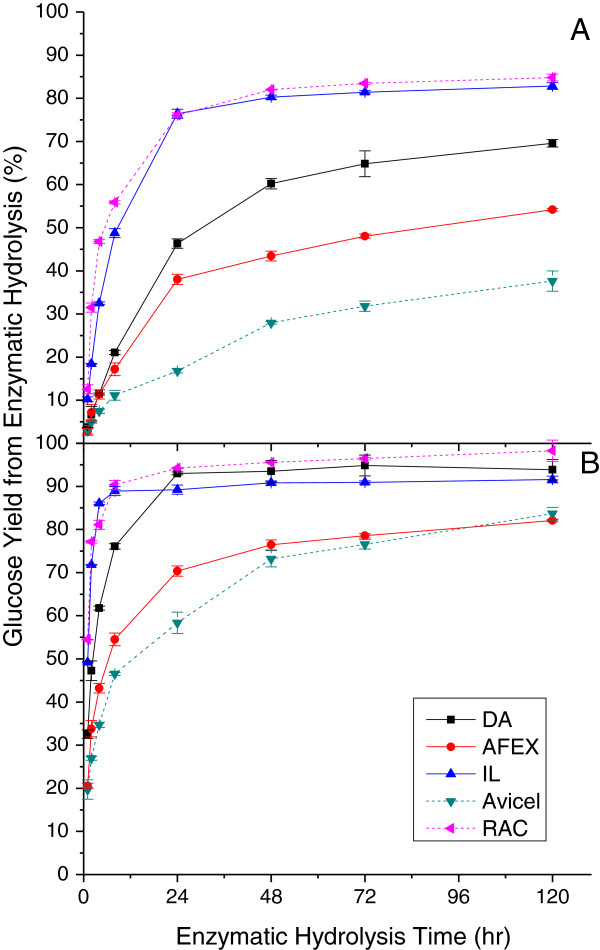
**Glucose yields in solution following enzymatic hydrolysis of DA, AFEX, and IL pretreated corn stover solids, Avicel cellulose, and RAC.** Enzyme loadings of **(A)** 3 mg and **(B)** 30 mg protein/g glucan in the raw corn stover. AFEX, ammonia fiber expansion; DA, dilute sulfuric acid; IL, ionic liquid; RAC, regenerated amorphous cellulose.

As shown in Figure 1, glucose yields from hydrolysis of DA and AFEX pretreated corn stover were rapid in the first 8 hours and then slowed down considerably to almost level off after 72 hours at both 3 and 30 mg protein/g glucan loadings. As expected, glucose yields were always higher at the higher enzyme loading, particularly for AFEX and DA pretreated solids. Corn stover solids from IL pretreatment had greater initial glucose yields compared to either DA or AFEX pretreated corn stover and nearly reached its maximum glucose yield in only 8 hours. Adding more enzyme increased glucose yields from IL pretreated solids less than from the other two pretreatments because the yields were already high for IL solids.

**Figure 2:**
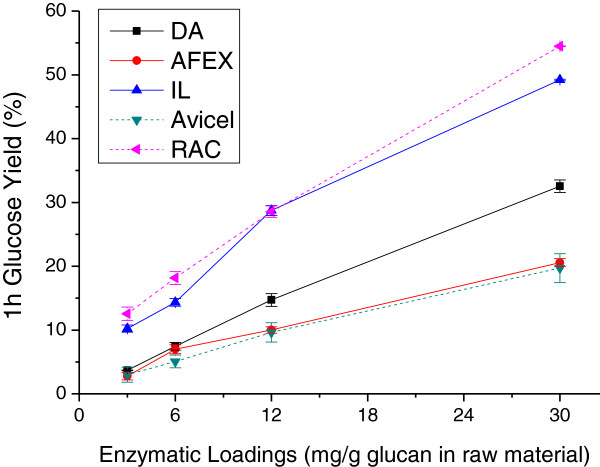
**Effect of enzyme loadings on the 1-hour glucose yields from enzymatic hydrolysis of DA, AFEX, and IL pretreated corn stover solids, Avicel cellulose, and RAC.** AFEX, ammonia fiber expansion; DA, dilute sulfuric acid; IL, ionic liquid; RAC, regenerated amorphous cellulose.

**Figure 3:**
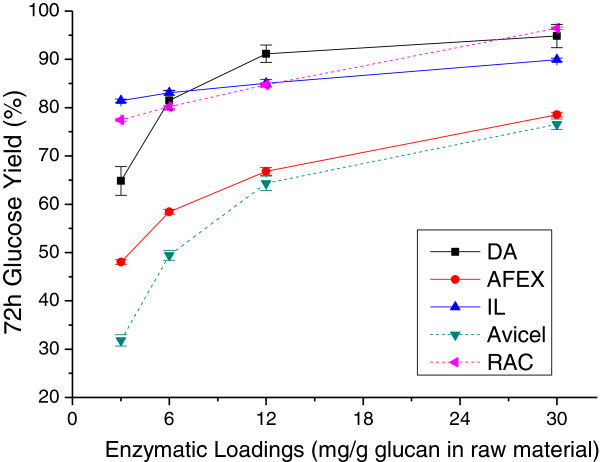
**Effect of enzyme loadings on the 72-hour glucose yields from enzymatic hydrolysis of DA, AFEX, and IL pretreated corn stover solids, Avicel cellulose, and RAC.** AFEX, ammonia fiber expansion; DA, dilute sulfuric acid; IL, ionic liquid; RAC, regenerated amorphous cellulose.

Figures 2 and 3 compare the 1-hour and 72-hour glucose yields from enzymatic hydrolysis at four enzyme loadings applied to solids produced from corn stover by the three pretreatments. Figure 2 shows that the 1-hour glucose yields of all substrates increased almost linearly with enzyme loading. Furthermore, IL pretreated corn stover solids displayed the highest 1-hour hydrolysis yield at all enzyme loadings followed by DA and then AFEX. Increasing the enzyme loading from 3 to 30 mg protein/g glucan increased glucose yields from IL pretreated solids by 40% in the first hour of hydrolysis. The 1-hour glucose yields from DA corn stover at the highest enzyme loading of 30 mg/g glucan were about six times higher than at the lowest enzyme loading of 3 mg/g glucan. The 1-hour glucose yield from solids from AFEX pretreatment was slightly lower compared to DA at the lowest enzyme loading but 10% lower at the high enzyme loading. Thus, the 1-hour glucose yield from AFEX pretreated corn stover did not benefit from higher enzyme loadings as much as solids from the other two pretreatments.

The 72-hour glucose yields from enzymatic hydrolysis of the pretreated solids were also compared for the three pretreatments at four enzyme loadings, as shown in Figure 3. Overall, higher enzyme loadings resulted in higher glucose yields, as expected, but the benefit of adding more enzymes varied with pretreatment. For example, as shown in Figure 3, when the enzyme loading was increased from 3 to 30 mg/g glucan in raw biomass, the 72-hour glucose yields for DA and AFEX corn stover increased by about 30% and 30.5%, respectively, while solids from IL pretreatment of corn stover had only about a 10% enhancement in glucose yield as it was already close to the maximum at the low enzyme loading. As seen in Figure 3, at 30 mg total protein for the optimized enzyme mixtures/g glucan in raw corn stover, 72-hour glucose yields from enzymatic hydrolysis were 93.8% for DA pretreated corn stover, 91.6% for IL, and 82.1% for AFEX. Avicel® and regenerated amorphous cellulose (RAC) model compounds realized about 42% and 16% increases in glucose yields, respectively, over the range of enzyme loadings applied.

#### Xylose monomer and oligomer yields

**Figure 4:**
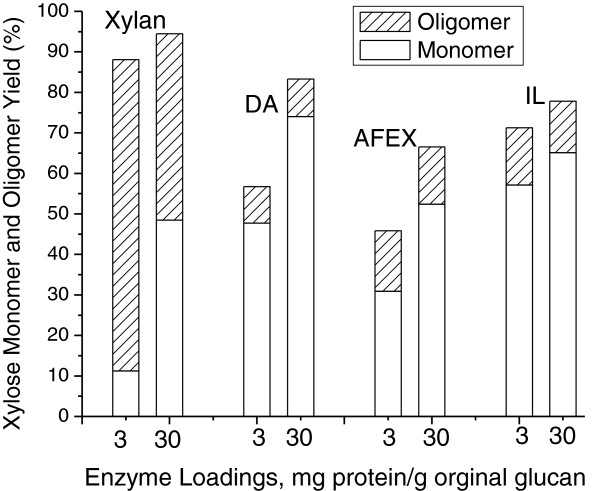
**Xylose monomer and oligomers yields from 72-hour enzymatic hydrolysis of DA, AFEX, and IL pretreated corn stover, and beechwood xylan.** Note only HTec2 was added to beechwood xylan. AFEX, ammonia fiber expansion; DA, dilute sulfuric acid; IL, ionic liquid.

The liquid samples from 4, 24, and 72 hours of enzymatic hydrolysis of solids produced by DA, AFEX, and IL pretreatments of corn stover were analyzed for longer chain length glucooligomers (> cellobiose) and xylooligomers by post-hydrolysis of liquid samples from pretreatment with 4% w/w DA at 121°C for 1 hour [41]. Figure 4 reports both xylose monomer and xylooligomer yields following 72 hours of enzymatic hydrolysis of solids produced by DA, AFEX, and IL pretreatments of corn stover at total enzyme protein loadings of 3 and 30 mg/g glucan in raw biomass for the same optimized enzyme mixtures as used to obtain glucose yields. Results from enzymatic hydrolysis of the model compound beechwood xylan are also included over a range of 3 to 30 mg of just HTec2 xylanase protein/g xylan. Figure 4 shows that about a quarter of the total xylose in solution was released as xylooligomers during hydrolysis for all pretreated solids and remained at about the same fraction of the total for both enzyme loadings for these three solids. However, a much higher fraction of the total xylose in solution was as oligomers for beechwood xylan, particularly at the lower enzyme loading, most likely due to low β-xylosidase and other accessory activities [37]. When comparing solubilized xylan yields from enzymatic hydrolysis of pretreated solids at the 10 g glucan/L employed for evaluating substrate breakdown by enzymes, it should be kept in mind that compositional variations in the substrates from each pretreatment led to quite different ratios of enzyme protein to xylan content. As shown in Figure 4, at an enzyme loading of 30 mg total protein/g glucan in raw corn stover, the total xylose monomer plus oligomer yields for solids produced from corn stover by DA, AFEX, and IL were 83%, 68%, and 77%, respectively, with oligomers contributing about 9%, 14%, and 12%, respectively, to these amounts.

#### Percentage of oligomers released during hydrolysis

**Figure 5:**
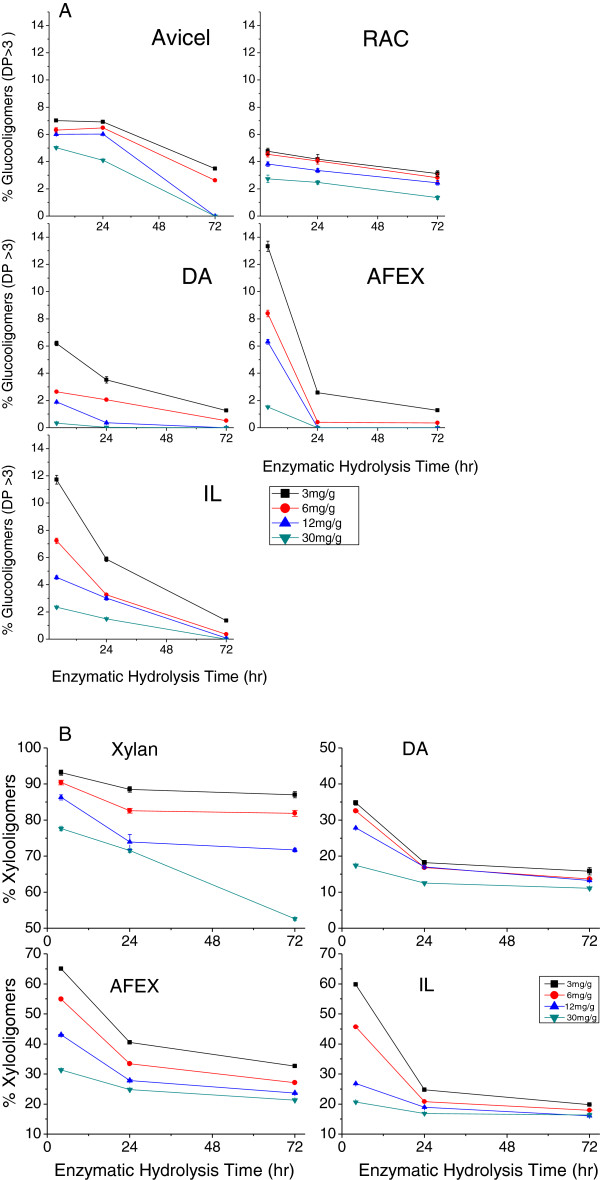
**Soluble glucooligomers (DP >3) and xylooligomers as a percentage of the total glucose and xylose in solution following 72 hours of hydrolysis at enzyme loadings of 3, 6, 12, and 30 mg/g glucan in raw biomass for Avicel, RAC, and beechwood xylan and DA, AFEX, and IL pretreated corn stover solids. (A)** Glucooligomers and **(B)** xylooligomers. AFEX, ammonia fiber expansion; DA, dilute sulfuric acid; DP, degree of polymerization; IL, ionic liquid; RAC, regenerated amorphous cellulose.

As shown in Figure 5A for glucooligomers and Figure 5B for xylooligomers, longer hydrolysis times and increased enzyme loadings both reduced the proportion of gluco- and xylooligomers. At the early stage of hydrolysis or for lower enzyme loadings, the maximum glucooligomer percentage was less than 13% for AFEX followed by 12% for IL and 6% for DA. Figure 5A suggests that application of mixtures of CTec2, HTec2, and Multifect Pectinase enzymes optimized individually for each pretreatment achieved essentially complete digestion of the glucan to monomers for all three pretreated materials at high enzyme loadings.

Figure 5B shows that hydrolysis of xylooligomers was less complete than for glucooligomers. For hydrolysis of AFEX and IL pretreated corn stover solids, approximately 20% or more of the total xylose in solution persisted as oligomers even after hydrolysis for 72 hours at an enzyme loading of 30 mg/g glucan in the raw material. Hydrolyzate from enzymatic hydrolysis of AFEX pretreated solids contained the highest amount of xylooligomers followed by IL and DA. Overall, enzymatic hydrolysis of DA pretreated corn stover released the lowest percentage of both glucooligomers and xylooligomers.

### Effect of xylan and lignin removal on enzymatic digestion

**Figure 6:**
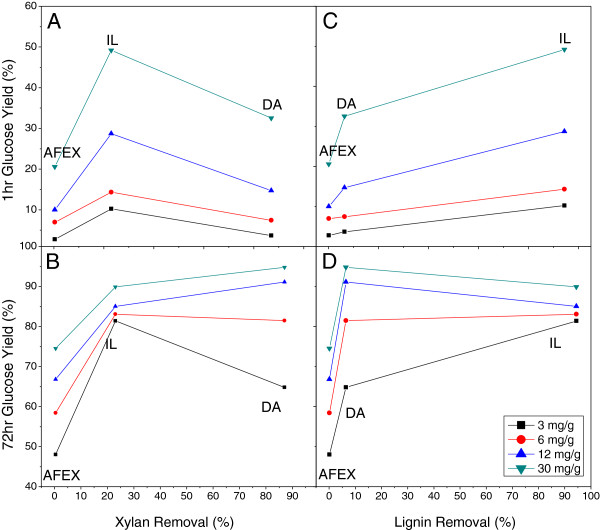
**The 1-hour and 72-hour glucose yields from enzymatic hydrolysis of DA, AFEX, and IL pretreated corn stover solids plotted against xylan removal and lignin removal at total enzyme loadings of 3, 6, 12, and 30 mg/g glucan in raw corn stover. (A, B)** Xylan removal and **(C, D)** lignin removal. AFEX, ammonia fiber expansion; DA, dilute sulfuric acid; IL, ionic liquid.

Figure 6 plots 1-hour and 72-hour glucose yields from enzymatic hydrolysis against lignin and xylan removal. Xylan removal has been reported to enhance glucan digestibility by improving cellulose accessibility [42] and/or reducing cellulase inhibition by xylooligomers produced from partial hydrolysis of xylan [43-45]. However, these results show that the 1-hour glucose yields did not follow a clear trend with xylan removal, and 72-hour yields only correlated with xylan removal at the two highest enzyme loadings.

From Figure 6, it is apparent that the 1-hour glucose yields correlated well with lignin removal at all enzyme loadings, while such a relationship was only apparent for 72-hour results at the lowest enzyme loading. This result is consistent with reports that lignin is one of the key biomass components impacting enzymatic digestion of cellulosic biomass [2,34]. Lignin is believed to not only hinder cellulose accessibility as a result of LCC linkages but also impact cellulase effectiveness by unproductive binding of enzymes [31,46,47].

**Figure 7:**
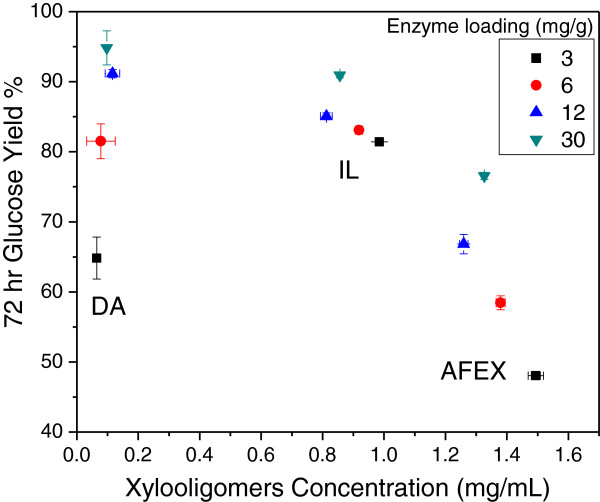
**The 72-hour glucose yields at enzyme loadings of 3, 6, 12, and 30 mg enzyme protein/g glucan in the raw corn stover plotted versus xylooligomer concentration measured during enzymatic hydrolysis of DA, AFEX, and IL pretreated corn stover.** AFEX, ammonia fiber expansion; DA, dilute sulfuric acid; IL, ionic liquid.

It has been shown that xylobiose and xylooligomers with higher DP strongly inhibit enzymatic hydrolysis of pure cellulose, pure xylan, and pretreated corn stover [43], and that xylooligomers were more inhibitory to cellulase than xylose or xylan for an equivalent amount of xylose or than equal molar amounts of glucose or cellobiose [44]. Figure 7 reports glucose yields against the concentration of xylooligomers in solution following enzymatic hydrolysis for 72 hours over the full range of enzyme loadings employed in this study, with the concentration of xylooligomers calculated on an equivalent xylose mass basis. First, these results show that DA solids released much less xylooligomers during enzymatic hydrolysis than solids from IL or AFEX. In addition, the xylooligomer concentrations remained virtually constant after 72 hours of enzymatic hydrolysis for all three pretreated solids. These results also show that xylooligomer concentrations had no clear effect on the trends in 72-hour glucose yields from enzymatic hydrolysis of the three pretreated solids. In fact, 72-hour glucose yields from enzymatic hydrolysis of DA and AFEX pretreated solids increased significantly with enzyme loading even though the xylooligomer concentration remained virtually the same for each. On the other hand, 72-hour glucose yields from IL pretreated solids were high and changed little despite having a xylooligomer concentration that was about two-thirds of that for enzymatic hydrolysis of AFEX pretreated solids. Therefore, other factors such as lignin removal, unproductive binding of enzyme, or cellulose structure appeared to have a greater effect on glucose yields.

### Comparison to results with model compounds

Two model compounds, microcrystalline Avicel cellulose and RAC, were enzymatically hydrolyzed as well. Figure 1 shows that glucose release was greatest from enzymatic hydrolysis of RAC and lowest from Avicel cellulose at the highest and lowest enzyme loadings applied in this study over almost the entire hydrolysis time. These trends are generally reinforced for the 1-hour and 72-hour glucose yields in Figures 2 and 3, respectively. In all cases, glucose yields from enzymatic hydrolysis of IL pretreated solids closely paralleled those with RAC. RAC was a highly homogeneous substrate with disrupted hydrogen bonds [48] and consequently had much higher glucose yields. Other than xylan and lignin removal, cellulose crystallinity is believed to be one of the major factors limiting cellulose enzymatic hydrolysis [48,49]. The high glucose yields from enzymatic hydrolysis of IL corn stover and RAC were probably due to reduction in crystalline cellulose content and/or altered cellulose structure previously shown for corn stover [36,50]. The rapid hydrolysis of amorphous substrate could be explained as a homogeneous reaction that enabled cleavage of all β-glycosidic bonds randomly, resulting in a rapid reduction of DP [48]. This result is consistent with an earlier hypothesis that lower crystallinity has a particularly significant influence on initial glucose yields from enzymatic hydrolysis [46].

### Possible causes of differences in sugar release

Based on the hydrolysis model reported by Zhang and Lynd [8], three processes occur simultaneously when enzymes act on insoluble cellulosic substrates: 1) chemical and physical changes in the solid residue; 2) primary hydrolysis in which the solid phase is hydrolyzed into soluble cellodextrins; and 3) secondary hydrolysis in which the soluble oligomers are further hydrolyzed into monomers. Given that the rate of primary hydrolysis is much slower than the rate of secondary hydrolysis, a substrate with greater primary hydrolysis sugar release should result in more rapid initial glucose release. The experimental observation of similar glucose yield patterns for IL pretreated corn stover and RAC solids suggest structural similarities. However, cellulose in corn stover from DA and AFEX pretreatment are expected to be more crystalline, while Avicel cellulose is known to be highly crystalline. This difference in crystallinity could at least partially explain why glucose yields from IL corn stover solids were higher than from the AFEX and DA pretreated solids and much higher than from Avicel, particularly at shorter times.

### Adsorption of CTec2 and HTec2 on pretreated corn stover

Enzyme adsorption onto the substrate is the primary step in enzymatic degradation of cellulose [9,51]. Cellulose accessibility to enzyme has long been recognized as an essential factor controlling enzymatic hydrolysis of cellulosic biomass [52-54], and enzymatic hydrolysis rates and yields are often claimed to be related to enzyme adsorption [42,52,53,55,56]. Adsorption parameters calculated for the Langmuir model, the maximum adsorption capacity σ and equilibrium constant K_d_, are summarized in Table 3. These results show substantial variances in enzyme adsorption with pretreatment type. AFEX corn stover had the lowest maximum enzyme adsorption capacity for both CTec2 and HTec2, while IL corn stover had the highest values for both. In line with this reasoning, the initial hydrolysis yields of the three pretreated substrates reported in Figures 1 and 2 followed the same trend as their maximum enzyme adsorption capacities for both CTec2 and HTec2. Thus, enzyme adsorption onto solids and their effectiveness are affected by pretreatment type and biomass composition, consistent with other published information [47,53,57].

**Table 3:** Maximum CTec2 and HTec2 adsorption capacities, equilibrium constants, and correlation coefficients for solids resulting from pretreatments of corn stover by DA, AFEX, and IL pretreatments

	**Pretreatment**		
	**DA**	**AFEX**	**IL**
**CTec2**			
Maximum adsorption capacity, σ (mg/g substrate)	139	111	190
Equilibrium constants, K_d_ (mg/mL)	1.88	0.19	1.43
R^2^	0.92	0.96	0.92
**HTec2**			
Maximum adsorption capacity, σ (mg/g substrate)	142	127	239
Equilibrium constants, K_d_ (mg/mL)	0.67	0.27	2.3
R^2^	0.97	0.95	0.98

## Conclusions

No single factor absolutely dominated early and longer-term glucose yields from enzymatic hydrolysis of solids from AFEX, DA, and IL pretreatments. The high initial hydrolysis yields from IL corn stover correlated with high lignin removal, high change in crystallinity, and high enzyme adsorption, and was very similar in pattern to results from enzymatic digestion of RAC solids. The final glucose yields did not follow a consistent trend with concentration of xylooligomers released from xylan during hydrolysis. IL pretreated corn stover showed the highest initial glucose yields at low enzyme loadings, while DA pretreated corn stover, which removed the most xylan, achieved the highest glucose yields at high enzyme loadings.

## Materials and methods

### Pretreated corn stover and model compounds

Corn stover was harvested in September 2008 at the Michigan State University Farms (East Lansing, MI, USA) from the corn hybrid NK 49-E3 (Syngenta, Basel, Switzerland), typical of that grown in the Great Lakes region. Solids resulting from pretreatment of the same source of corn stover were prepared by the collaboration partners of the Bioenergy Research Centers (BRCs), as follows: DA pretreatment by BESC at UCR, AFEX by GLBRC at Michigan State University, and IL by JBEI. Upon receipt, the AFEX and IL pretreated corn stover solids were immediately refrigerated at 4°C until further analysis. The pretreatment conditions summarized in Table 1 for all three pretreatments were selected based on highest total glucan plus xylan yields from both pretreatment and enzymatic digestion, but only the solids were employed in the enzymatic hydrolysis study reported here.

Pure cellulose (Avicel PH101, catalogue number 11365, lot number 1094627) was purchased from FMC Corporation (Philadelphia, PA, USA). RAC was prepared from Avicel PH101 according to the method reported by Zhang and coworkers [48]. Beechwood xylan (lot number BCBS8393V) was purchased from Sigma Chemicals (St Louis, MO, USA). Moisture content and compositional analysis of the corn stover solids and model compounds were determined according to the NREL LAP [58].

#### Enzymes

Cellic CTec2 (batch number VCNI0001) and Cellic HTec2 (batch number VHN0001) enzymes were generously provided by Novozymes North America, Inc., and Multifect Pectinase (batch number 4861295753) was from DuPont Industrial Biosciences. Table 4 shows the enzyme protein concentrations determined by the Kjeldahl method [59], with the nitrogen factor (NF) calculated by Equation 1:

(1) NF=%protein/%nitrogen

in which the percentage of protein was calculated as:

(2) $$\begin{array}{l} \%\text{protein}=\text{protein}\;\text{content}\;\left(\text{mg}/\text{mL}\right)\\ \;/\text{solid}\;\text{concentration}\;\left(\text{mg}/\mathrm{mL}\right) \end{array}$$

**Table 4:** Enzyme nomenclatures, descriptions, protein concentrations, and nitrogen factors

**Enzyme**	**Description**	**Protein concentration (mg/mL)**	**Nitrogen factor**
Cellic® CTec2	Blend of cellulase, high level of β-glucosidase, and hemicellulases	138	6.09
Cellic® HTec2	Blend of hemicellulases and cellulase background	157	6.58
Multifect® Pectinase	Pectinase, cellulase, and hemicellulases	72	-

The nitrogen content was determined by following a previously described method [60]. The solids content of the enzyme solution was determined according to the NREL LAP [61].

### Enzymatic hydrolysis

In accordance with the NREL LAP [38], enzymatic hydrolysis was conducted in triplicate at a solids loading corresponding to 1% (w/w) glucan in 0.05 M citrate buffer (pH = 4.9) containing 10 mg/mL sodium azide in 50 mL Erlenmeyer flasks. The slurries were incubated at 50°C for 120 hours in a shaker incubator (Multitron Infors-HT, ATR Biotech, Laurel, MD, USA) at 150 rpm. Enzyme loadings were 3, 6, 12, and 30 mg of total protein/g glucan in the raw biomass. Enzyme combinations of Cellic CTec2, Cellic HTec2, and Multifect Pectinase to achieve maximum sugar release for solids from DA, AFEX, and IL pretreatments were determined by GLBRC using their novel high-throughput microplate hydrolysis method [39], and are shown in Table 4.

Hydrolysis samples were collected at 1, 2, 4, 8, 24, 48, 72, and 120 hours. To determine the amount of sugar generated from enzymatic hydrolysis, 400 μL samples were drawn, filtered through 0.2 μm nylon filter vials (Alltech Associates Inc., Deerfield, IL, USA), pipetted into 500 μL polyethylene HPLC vials, and then stored at 4°C until analysis. Glucan to glucose and xylan to xylose hydrolysis yields were calculated according to the following two equations, respectively:

(3) $$\begin{array}{l} \%\text{Glucuse}\;\text{yield}=100\times \left(\left(\mathrm{GH}\left(g\right)+\mathrm{CB}\left(g\right)\times 1.503\right)\right)/\\ \;\left(1.111*\mathrm{GP}\left(g\right)\right) \end{array}$$

(4) $$\%\text{Xylose}\;\text{yield}=100\times \mathrm{XH}\left(g\right)/\left(1.136*\mathrm{XP}\left(g\right)\right)$$

in which GH, CB, and XH are the measured masses of glucose, cellobiose, and xylose released from enzymatic hydrolysis; GP and XP are the masses of glucan and xylan available in the pretreated biomass; and the factors 1.111, 1.136, and 1.053 account for the mass gained during hydrolysis of glucan to glucose, xylan to xylose, and cellobiose to glucose, respectively.

### Basis for enzyme protein loading per gram of glucan in raw biomass

Consistent with the approach used by our team in prior research at the recommendation of the Consortium for Applied Fundamentals and Innovation (CAFI) Advisory Board, enzyme loadings for all enzymatic digestion experiments in this study were based on glucan content in the original raw material [1,35]. Loading enzyme based on glucan content in raw biomass better represents the cost of enzyme per amount of potential ethanol, and also benefits removing more glucan in pretreatment with more enzyme per unit of glucan left in the solids to enzymatic hydrolysis. This comparison is particularly important for enzymatic hydrolysis at commercially viable low enzyme loadings. Because of the differences in glucan removal by the three pretreatments of interest here, the enzyme loadings per gram of glucan in the pretreated biomass solids varied as shown in Table 5. The result is that solids from DA and IL pretreatments had higher enzyme loadings per gram of glucan in the pretreated solids compared to solids from AFEX.

**Table 5:** Glucan recovery in solids following pretreatment and enzyme loadings for hydrolysis of pretreated solids based on glucan content in raw corn stover

**Pretreatment**	**Glucan yield (%)**	**Enzyme loading (mg protein/g glucan in raw)**	**Enzyme loading (mg/g glucan in pretreated)**
Dilute acid	87	30	34
AFEX	100		30
Ionic liquid	90		33

Glucan yield in solids = Glucan in pretreated biomass (g)/ Glucan in the starting material (g).

Enzyme loading per g glucan in pretreated biomass = Enzyme loading per g glucan in raw/ glucan yield in solids.

### Estimation of the amounts of oligomers

To determine the total amount of glucose and xylose oligomers generated by enzymatic hydrolysis, liquid samples following enzymatic hydrolysis for 4, 24, and 72 hours were subjected to post-hydrolysis according to the NREL LAP [41]. In particular, slurries after enzymatic hydrolysis were centrifuged to separate solids from liquid. Then, the liquid was incubated for 1 hour with 4% sulfuric acid at 121°C in an autoclave (model HA300MII; Hirayama Manufacturing Corporation, Saitama, Japan) along with sugar recovery standards. It is important to note that post-hydrolysis was carried out in 1.5 mL high recovery glass HPLC vials (Agilent, Santa Clara, CA, USA) and scaled down to 1 mL reaction volume instead of applying the conventional method in 125 mL pressure bottles with 5 to 20 mL liquid [62]. Following post-hydrolysis, about 400 μL samples were withdrawn, pipetted into 500 μL polyethylene HPLC vials, and kept at 4°C or frozen at −20°C until further analysis. From this information, the percentage of the total glucose in solution that was glucooligomers with a DP > cellobiose, G_3+_, was calculated by:

(5) %G3+=100×GH'g−GHg−1.053×CBg/GH'g

X_2+,_ the percentage yield of xylooligomers containing two or more xylose units, was calculated as:

(6) %X2+=100×XH'g−XHg/XPg∗1.136

In addition, the percentage of the total xylose in solution that was oligomers containing two or more xylose units was calculated as:

(7) %XS2+=100×XH'g−XHg/XH'g

GH’ and XH’ represent the masses of glucose and xylose measured after post-hydrolysis and adjusting for losses by the sugar recovery standard [41].

### Sugar analysis

Samples along with appropriate calibration standards were run on a Waters Alliance HPLC system (Model e-2695; Waters Corporation, Milford, MA, USA) employing an Aminex® HPX-87H column (Bio-Rad Laboratories, Life Science Research, Hercules, CA, USA). Samples were processed at an eluent (5 mM sulfuric acid) flow rate of 0.60 mL/min using a refractive index (RI) detector (Model 2414; Waters Corporation). The chromatograms were recorded and processed with Empower® 2 software (Waters Corporation).

### Enzyme adsorption

Adsorption experiments were performed at 4°C in 0.05 M citrate buffer (pH = 4.8 ± 0.2) in 15 mL test tubes (catalogue number 430055; Thermo Fisher Scientific Inc., Waltham, MA, USA) with a biomass loading to achieve 1% w/w glucan with enzyme loadings of 0 to 2,000 mg protein/g glucan (0 to 20 mg protein/mL). The tubes containing biomass slurry and enzyme proteins were mounted on a variable speed rugged rotator (Glass-Col, LLC, Terre Haute, IN, USA) and equilibrated for 6 hours at 40 rpm. Following equilibration, the tubes were centrifuged (model Allegra X-15R; Beckman Coulter, Fullerton, CA, USA) for 15 minutes at 3,500 rpm for solid–liquid separation, the liquid was decanted, and the tubes dried overnight at 105°C. The adsorbed protein amount was directly determined by the nitrogen factor method described above [60]. The nitrogen content of the dried and homogenized biomass solids was measured using a Flash EATM 112 N/Protein plus CHNS/O Analyzer (CE Elantech, Lakewood, NJ, USA) with atropine as a standard (catalogue number 33835210; CE Elantech). Adsorption data was non-linearly fit to a Langmuir model according to Equation 8 [7,63]:

(8) $$\left[\mathrm{CE}\right]=\frac{\sigma \left[S_{t}\right]\left[E_{f}\right]}{K_{d}+\left[E_{f}\right]}$$

in which [CE] is the amount of adsorbed enzyme in mg/mL, [E_f_] is the free enzyme concentration in mg/mL, σ is the maximum adsorption capacity in mg/mg substrate, [S_t_] is the substrate concentration mg/mL, and K_d_ is the equilibrium constant equal to [C] [E]/[CE].

## Abbreviations

AFEX: Ammonia fiber expansion; BESC: BioEnergy Science Center; BRCs: Bioenergy Research Centers; CAFI: Consortium for Applied Fundamentals and Innovation; DA: Dilute sulfuric acid; DP: Degree of polymerization; GLBRC: Great Lakes Bioenergy Research Center; HPLC: High performance liquid chromatography; IL: Ionic liquid; JBEI: Joint BioEnergy Institute; LAP: Laboratory Analytical Procedure; LCC: Lignin-carbohydrate complex; NREL: National Renewable Energy Laboratory; RAC: Regenerated amorphous cellulose; RI: Refractive index; UCR: University of California, Riverside.

## Competing interests

The authors declare that CEW is a cofounder of Mascoma Corporation (Lebanon, NH, USA) and former chair of their Scientific Advisory Board. CEW no longer works with Mascoma. In addition, CEW is founding Editor-in-Chief of *Biotechnology for Biofuels*. The other authors declare that they have no competing interests.

## Authors’ contributions

XG performed pretreatment of corn stover with dilute acid, all enzymatic hydrolysis experiments and characterization work reported here, prepared the initial manuscript, and undertook final assembly of the revised paper. CEW developed the initial enzymatic hydrolysis and dilute acid pretreatment experimental design, supervised the work, revised the manuscript, figures, tables, and preparation of correspondence, and approved the final manuscript. RK assisted in the design of experiments, reviewed the results, and prepared and reviewed the manuscript. BED initiated the collaboration, arrangements for supply of corn stover and AFEX pretreated solids, and reviewed the manuscript. VB supplied the corn stover, provision of AFEX pretreated materials, and reviewed the manuscript. BAS supplied the IL pretreated materials and reviewed the manuscript. SS supplied the IL pretreated materials and reviewed the manuscript. All authors read and approved the final manuscript.
